# Nebuchadnezzar's Feast

**Published:** 1893-04-15

**Authors:** 


### Nebuchadnezzar's Feast.

We have received a volume of Essays on Vegetarianism which we have read with mingled irritation and depression. Without being vegetarian either in principle or practice, we think that vegetarianism is doing good service to the cookery of England. Hitherto we have lagged behind all the nations in our use of vegetables. What do we do with them? Boil them to insipidity and throw away the water in which a large proportion of the salts which form their most valuable constituents are held in solution. As for fruit, an occasional pie of rhubarb or apple, a careless trifling with strawberries at afternoon tea, and after dinner a couple of nuts eaten solely to give relish to the wine, sum up all that many of us indulge in. Fruit is, indeed, too much regarded as an indulgence, a luxury; too little as an important part of our daily food. Both these errors vegetarians are doing much to cure—or were so doing; for we speak of what we believed to be the vegetarian creed before we read Mr. A. F. Hills' "Essays on Vegetarianism."

A flippant journalist once observed that vegetarians might be divided into the same three classes as Churchmen—the High, the Low, and the Broad. Mr. Hills does not belong to the Broad vegetarian communion, which admits milk, butter, and eggs, and even winks at the sacrifice of an occasional fish. (In truth, we also might be found among these prophets, and would vegetate gladly on cucumber—with a slice of salmon to accompany it.) But to Mr. Hills these weaker brethren are *anathema maranatha.* He condemns even the Low or cabbage-loving variety of the sect. We thought, indeed, that we had points of sympathy with him when we found him condemning the vegetarian cookery of the restaurants where "sodden pottage alternates with stodgy porridge." We also had found such fare unappetising, but we were not prepared for the alternative. That alternative is—uncooked grain. The ideal man takes his food "untouched by fire, unscathed by flame," and from his point of view roast chicken and boiled asparagus are equally criminal. To the mass even of vegetarians such a rule must appear at best a counsel of perfection, not to be followed save by the select of the elect—speaking of vegetarians one dare not say the *crème de la crème;* to the rest of the world it will seem a miracle of folly. Nay, to the fastidious it will seem, when they read a chapter on the virtues of uncooked cereals prefaced by the quotation—

> Nearer, my God, to Thee,
> Nearer to Thee,
> E'en though it be a cross
> That raiseth me, that the well-meaning author goes perilously near to profanity.

Let us state once for all, and as emphatically as we can, that the idea of our food being a cross is not merely ridiculous, but wicked. Eating, like every other natural exercise, should be pleasant; it is, within limits which define themselves, a legitimate pleasure. A healthy man enjoys his meals; an unhealthy man does not, though you ransack earth, sea, and sky to please his palate. We have no respect for the glutton and the gourmand, any more than for the drunkard or the debauchee, or any other who perverts a natural appetite into an unnatural craving; but it is well to remember that these natural appetites are divinely implanted and ordained; and that the mere mortifying of the flesh has no merit in itself. "A hungry man," says the old proverb, "is an angry man." The truth of that saying every housekeeper has proved; and we will need something more than has yet been told us to be convinced that a hungry man is, because of his hunger, or because he has eaten only tasteless food of a limited range, a more religious man than his fellows.

Of course, the usual argument is used—this food, these raw grains varied by a few nuts, is the natural food of man. On every subject under the sun this argument of nature's law is brought up. One would just like to ask these eminent *naturalistes* if they think that any European, American, or Asiatic human being leads the life of a natural man. Some of the natives of Africa may still be regarded as natural in their ways; but most even of these will degenerate into civilisation.

"When wild in woods the noble savage ran,"

it is highly probable that he lived on grains, roots, and fruits, "untouched by fire, unscathed by flame," because he could do no other. When the Prometheus of his nation taught him how to produce fire, it is likely enough he took to cookery and a flesh diet; if, indeed, he was not carnivorous before he had a fire, like those exceedingly natural specimens of the human race, the Aleuts of the Pribyalov Islands, who consume seal-blubber raw. Even if it could be proved that uncooked cereals were the natural food of man, we feel no more compelled to adopt it in its lean entirety than to give up the conventional habit of wearing clothing, or to forsake our comfortable houses and soft beds to roam in forests or to sleep in caves. The natural man did not study medicine, nor practice at the bar, nor struggle with the difficulties of Hebrew texts, nor was worried by "business in the city." Circumstances have modified our natural condition in every way; in some ways, perhaps, for the worse, but on the whole for the better. Our food, it may be, has changed with the rest, but it is as injudicious, in many cases as impossible, to revert to the noble savage's limited menu as to some of his other customs.

The plea of nature, then, avails little with us; but if Mr. Hills can prove that the Nebuchadnezzar's feast to which he invites us is more wholesome than our mixed diet, we will come to his table, even though our corrupt palates long for the masterpieces of the artist of the kitchen. He says that it is, but he does not prove it, save by these repeated quotations from the hymnal; and the cases of two vegetarian babies, who did not thrive upon a diet of nuts, but improved when raw wheat was added to their supplies, are not encouraging. Poor vegetarian babies, taken from their mothers' breasts, and set to struggle with nuts! If we are, indeed, to go by nature, will Mr. Hills pretend that nature ever created a human baby that was instinctively vegetarian? One would fain introduce into these homes, not nuts and grains, but a kindly cow and the gravy from a piece of juicy meat; and if Mr. Hills will test results by a baby-show, we are prepared to stand or fall by the size, colour, firmness, and what Artemus Ward termed, the "fiting wate," of the non-vegetarian child. If adults will experiment upon their digestions they are free to do so, but let them leave to helpless infancy its abundance of milk, and administer the starch-foods which it needs in some palatable form. Let it have fruit and sugar *à discretion*, but let its mainstay be milk—and that not the milk of the cocoanut, which no one, we fancy, ever paralleled to human kindness.

It is, indeed, infinitely to be regretted that such a book as this was ever written. Vegetarians can do useful work if they will teach us how to cook vegetables in the most nutritive and appetising way; if they will interest themselves in fruit culture, and bring the price of what are now luxuries within the reach of all; if they will, indeed, show us how to save our purses without impairing our digestion or weakening our physical and mental powers; but to do this they must preach a gospel of moderation. That we have not yet reached the perfect way of living we are most willing to admit; that we may learn to make a wider and wiser use of vegetable products we gladly own; but that is another matter from adopting vegetarianism. And the extreme vegetarianism which would confine our diet to nuts and uncooked grains must, if it would win converts, show better physical results than any vegetarian we have yet seen, and stronger mental qualities than Mr. Hills' book betrays.

